# Integrating findings of traditional medicine with modern pharmaceutical research: the potential role of linked open data

**DOI:** 10.1186/1749-8546-5-43

**Published:** 2010-12-17

**Authors:** Matthias Samwald, Michel Dumontier, Jun Zhao, Joanne S Luciano, Michael Scott Marshall, Kei Cheung

**Affiliations:** 1 Digital Enterprise Research Institute, National University of Ireland Galway, IDA Business Park, Lower Dangan, Galway, Ireland; 2 Information Retrieval Facility, Donau City Straße 1, 1220 Vienna, Austria; 3 Department of Biology, Institute of Biochemistry, School of Computer Science, Carleton University, 1125 Colonel By Drive, Ottawa, Ontario K1S 5B6, Canada; 4 Department of Zoology, University of Oxford, The Tinbergen Building, South Parks Road, Oxford, OX1 3PS, UK; 5 Tetherless World Constellation, Rensselaer Polytechnic Institute, Winslow Building, Room 2143, 110 8th Street, Troy, NY 12180, USA; 6 Informatics Institute, University of Amsterdam, Kruislaan 403, 1098 SJ Amsterdam, The Netherlands; 7 Center for Medical Informatics, Yale University School of Medicine, 300 George Street, New Haven, CT 06511, USA

## Abstract

One of the biggest obstacles to progress in modern pharmaceutical research is the difficulty of integrating all available research findings into effective therapies for humans. Studies of traditionally used pharmacologically active plants and other substances in traditional medicines may be valuable sources of previously unknown compounds with therapeutic actions. However, the integration of findings from traditional medicines can be fraught with difficulties and misunderstandings. This article proposes an approach to use linked open data and Semantic Web technologies to address the heterogeneous data integration problem. The approach is based on our initial experiences with implementing an integrated *web of data *for a selected use-case, i.e., the identification of plant species used in Chinese medicine that indicate potential antidepressant activities.

## Background

Ethnopharmacological findings are scattered over a multitude of publications and databases and are not well connected to other biomedical databases. As a result, the utility of these sources as knowledge resources are severely limited, which creates a further obstacle for modern day e-science research, which relies heavily on multiple heterogeneous data sources. Semantic technologies and standards, such as the Resource Description Framework (RDF) [1] and the Web Ontology Language (OWL) [2] provide technology that has potential to be used to help tackle the problem [3]. In recent years, relevant databases have been converted their data into the RDF/OWL format. This effort is exemplified by DartGrid, a toolkit for exposing relational datasets in RDF/OWL format [4]. A large-scale e-science infrastructure of datasets and ontologies for Chinese medicine was developed [5-7]. Unfortunately, the public accessibility to many of these resources is limited. This article proposes an alternate approach, using linked open data and Semantic Web technologies to address the heterogeneous data integration problem.

### Semantic Web approach

We investigated the usefulness of openly available RDF/OWL tools and datasets to find evidence for pharmaceutical compounds from Chinese medicine that may treat depressive disorders or serve as lead compounds for the future pharmaceutical drug development. The reasons for choosing a psychological disorder were two-fold. Firstly, the development of traditional medicines such as Chinese medicine was mainly guided by symptomatological and introspective observations without the need for sophisticated experimental methods available only to modern medicine. Mental conditions, such as depression, are amenable to these kinds of phenomenological observations. It is possible to use traditional medicines to identify the source of pharmacological compounds that may otherwise be missed by modern rational drug design. Secondly, the conceptualization of mental conditions is diverse across different eras and different cultures. For example, there seems to be no one-to-one equivalent to the concept of 'depressive disorder' in Chinese medicine. Instead, the symptoms of depression [8] match the symptoms associated with several major Chinese medicine classifications (Table 1) [9]. The use of semantic technologies may help bridge these gaps by making the meaning and interrelations of various concepts more explicit and facilitating the integration of heterogeneous data sources.

Based on these considerations, we explored current semantic resources and linked data technologies in order to identify their potential for improving the integration of findings from traditional medicines into modern pharmaceutical research. By centering this exploration on a concrete use-case, we aim to identify possible challenges using these technologies in practice-oriented settings.

**Table 1:** Chinese medicine categories with potential relevance for depressive disorders (adapted from 9)

TCM category	Indications	Examples of representative plants
*Shen *(Mind)	palpitations, anxiety, insomnia	*Zizyphus spinosa *Hu, *Platycladus orientalis *Franco, *Albizia julibrissin *Durazz.
Tonify Qi	lethargy, weakness, poor appetite, weak voice, pale complexion, breathlessness, immunodeficiency	*Panax ginseng *C. A. Mey, *Codonopsis pilosula *Nannf., *Astragalus propinquus *Schischkin, *Atractylodes *Koidz., *Glycyrrhiza spp., Dioscorea opposita *Thunb.
Tonify Yang	systemic exhaustion, fear of cold, cold extremities, withdrawal, sore and weak lower back, slow and deep pulse	*Cistanche deserticola *Ma, *Epimedium grandiflorum *Morr, *Psoralea corylifolia *L., *Alpinia oxyphylla *Miq., *Eucommia ulmoides *Oliver, *Dipsacus asper *Wall, *Morinda citrifolia L., Cnidium monnieri *L.
Phlegm (Heart)	delirium, seizure, coma, various psychiatric conditions (such as bipolar depression)	*Polygala tenuifolia *Willdenow, *Liquidambar orientalis *Miller, *Acorus gramineus *Sol.

**Figure 1:**
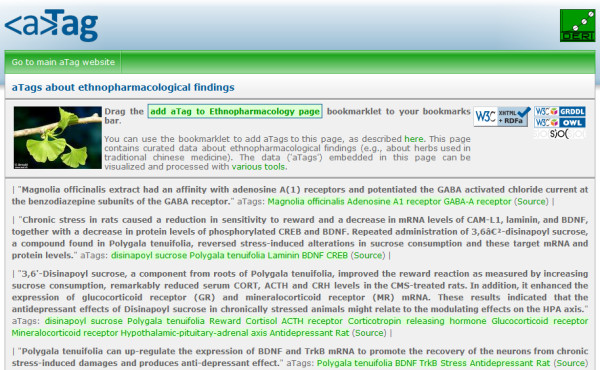
**An interactive web page for collecting curated statements from biomedical literature, annotated with entities from DBpedia**. The structured RDF data is embedded inside the webpage based on the RDFa standard.

As a starting point, we set up an interactive web page (Figure 1) [10] designed for the participants of the pilot project to collect curated statements from biomedical literature and annotate statements with entities from DBpedia [11], a large and comprehensive linked data resource derived from Wikipedia. This functionality was based on using associative tags (aTags) [12], the RDFa standard [13] and related tools that are described below. Through this annotation process, evidence for potential antidepressant activity of the representative plant species was collected from NCBI PubMed [14]. In total, 76 assertions were encoded in this manner. In addition to searching for documentation supporting antidepressant effects of these plants, we conducted a separate PubMed search for documentation on Chinese herbs associated with antidepressant effects.

The use of semantic annotations added practical value to the manually curated dataset we produced. Recently, TCMGeneDIT [15], a database of facts extracted from literature indicating associations between Chinese medicines, genes, diseases, effects and ingredients, was converted to RDF [16,17]. Since the RDF version of TCMGeneDIT contains a mapping to DBpedia, the manually curated aTags and the TCMGeneDIT dataset are semantically interlinked through their shared DBpedia identifiers, thereby demonstrating the potential of linked data technologies.

In addition to the data from traditional medicines, we generated aTags about pharmacogenomic findings associated with approved antidepressant pharmaceuticals [18] in order to relate and compare between traditional medicines and approved pharmaceuticals. The aTags were generated from known associations between gene variants, side effects and outcomes arising from drug treatments of depression. Relevant articles were initially identified by curators at the PharmGKB database [19] to identify articles about a pharmacogenomic association in the treatment of depression. Gene variants, side effects and clinical outcomes were curated from a subset of these articles and added to an ontology-driven knowledge base that extended the PharmGKB data in RDF format.

**Figure 2:**
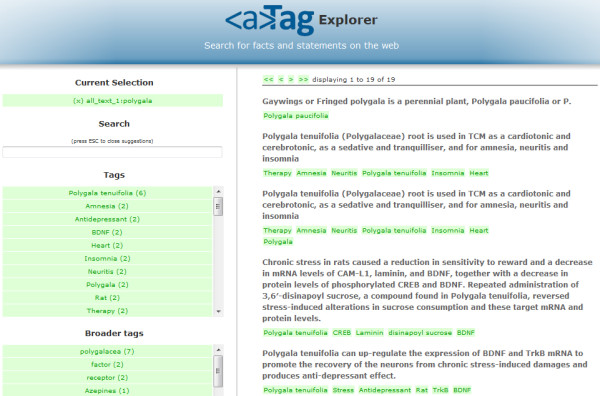
**The aTag explorer enables full-text search and faceted browsing of scientific statements encoded as aTags**. Since each aTag is annotated with entities from taxonomies/ontologies, it is possible to filter search results based on the entities that were used for annotation, as well as the broader concepts/superclasses of these entities.

After the creation and interlinking of the structured data described above, we analyzed the data in order to characterize the antidepressant activities of selected plant species by browsing the aggregated datasets with the aTag Explorer (Figure 2) [20]. The aTag Explorer is a web interface for faceted searching and browsing of aTags on the web. The RDF was loaded into the Health Care and Life Science Knowledge Base [21] to make it publically accessible for querying through a SPARQL endpoint. In the aTag Explorer and Knowledge Base, the scientific statements generated through manual curation may be queried alongside with hundreds of thousands of other statements derived from biomedical abstracts and structured databases.

### Preliminary results and evaluation

We identified several plant species whose potential antidepressant action was recorded in the Chinese medicine literature. The following text focuses on *Polygala tenuifolia*, *Magnolia officinalis *and *Albizia julibrissin*,three medicinal plants currently not known to possess activities related to the central nervous system.

### Relevant information in RDF/OWL resources

A search using Sindice [22] revealed no useful RDF/OWL data about these three plants apart from the manually curated data created by the authors of this article and the general information provided by DBPedia. Targeted queries in the linked data representations [23] of DrugBank [24,25] and Clinicaltrials.gov [26] found no information about the medical use of these three plants. They have not been tested in a controlled clinical trial.

We found the RDF version of TCMGeneDIT to contain data for two of the three plants, namely *Polygala tenuifolia and Magnolia officinalis*. Since the RDF version of TCMGeneDIT contains a map to DBpedia, the manually curated aTags and the TCMGeneDIT dataset are semantically interoperable through shared DBpedia identifiers.

### Examples of relevant pharmacological findings

Below we list examples of relevant pharmacological findings for each plant captured in the RDF/OWL resources we investigated.

*Polygala tenuifolia *(DBpedia identifier 'http://dbpedia.org/resource/Polygala_tenuifolia') is one of the 50 'fundamental herbs' used in Chinese medicine. Used for conditions such as delirium, seizure, coma and various psychiatric conditions, *Polygala tenuifolia *is associated with the 'Phlegm (Heart)' category in traditional Chinese medicine (TCM). According to DBpedia, however, it is mainly used as an expectorant. The RDF version of TCMGeneDIT contains several references for treatment effects, namely 'antipsychotic', 'cholinergic', 'therapeutic' and, seemingly contradictive, both 'antiinflamatory' and 'inflammatory'. References to antidepressant activity are lacking in TCMGeneDIT (and this is true for all of the plants presented here). The manually curated aTag dataset contains several curated statements from PubMed abstracts that clearly indicate an antidepressant action of *Polygala tenuifolia *and indicate that 3,6'-disinapoyl sucrose is the main compound responsible for these effects. These data suggest several interesting mechanisms of action behind these antidepressant effects, namely reduction of stress hormone levels, upregulation of neurotrophic factors and increased neuronal plasticity and neurogenesis [27,28].

*Magnolia officinalis *(DBpedia identifier 'http://dbpedia.org/resource/Magnolia_officinalis') is a widely known ornamental tree with a long history of medical use. The manually curated aTags about *Magnolia officinalis *point to several publications describing anxiolytic and antidepressant effects of *Magnolia officinalis *extracts [29,30]. Some potential mechanisms of action recorded in the curated dataset are modulation of GABA and adenosine receptors [31] as well as neurotrophic activity [32]. The main active ingredients responsible for these effects are Honokiol, Magnolol and related compounds.

The bark and flowers of *Albizia julibrissin *(DBpedia identifier 'http://dbpedia.org/resource/Albizia_julibrissin') are used in Chinese medicine. Associated with symptoms such as palpitations, anxiety and insomnia, *Albizia julibrissin *is classified under the '*Shen *(Mind)' category in TCM. A potential mechanism of action described in the literature is the general modulation of the serotonin system, especially modulation of 5-HT1 receptors. The connection between 5-HT1 receptors and antidepressant response was also found in aTags extracted from PubMed conclusion sections.

### How helpful are currently available semantic resources?

Several plants showing promising neurochemical and behavioral effects were identified and further characterized with semantic technologies. Most of these plants are obscure to the medical community outside Chinese medicine.

For researchers without a strong background in Chinese medicine, the categorization of diseases, symptoms and indications according to Chinese medicine theory can be misleading and confusing. For example, *Polygala tenuifolia*, one of the most promising plants with potential antidepressant activities according to PubMed abstracts, is found in the 'Phlegm (Heart)' category. Furthermore, the placement in a certain Chinese medicine category did not appear to be a reliable predictor of pharmacological activities in PubMed abstracts. This situation may be improved by a mapping between Chinese medicine classes and associated scientific categorizations of diseases, symptoms and indications, possibly formalized as an OWL ontology.

Increased reliance on well-structured consensus taxonomies with explicit semantics not only facilitates pharmacological research, but also helps prevent serious harm to patients by decreasing the probability of misunderstandings and errors in the formulation and prescription of herbal remedies. For instance, over a hundred cases of severe renal failure caused by aristolochic acids were reported in Europe [33] as a result of herbal mixtures erroneously containing the poisonous plant *Aristolochia fangchi*. The reason for this error was that some plant species from different regions of China have very similar names. For example, *Fangji *refers to two different plants, *Stephania tetrandra *(*Hanfangji*), which is the correct ingredient for the herbal mixture, and *Aristolochia fangchi *(*Guangfangji*), which contains highly nephrotoxic and carcinogenic aristolochic acids. A simple taxonomy or ontology of these pharmaceutical ingredients may help reduce such human errors.

While potential antidepressant activities are clearly described in literature, the TCMGeneDIT database and its RDF representation did not contain such data, underlining the well-known fact that the automated extraction of structured data from biomedical texts cannot be achieved with perfect recall and that manual curation is still a necessity to turn unstructured biomedical literature into structured data.

As expected, the manual curation of scientific statements in literature proved to be a time-consuming process, but manual curation is in many cases indispensable due to the limited availability of structured databases. While several databases for Chinese medicine exist [34], they are not publicly available and thus could not be integrated into the interlinked data structure we created. The unified Chinese medical language system UTCMLS [6], a large ontology/taxonomy for Chinese medicine, was not publicly available at the time of preparing this manuscript. It would be a significant gain for the research community if these databases were made publicly accessible.

RDF stores have been known to have performance issues, however, both performance and reliability of RDF stores has steadily improved in the past few years and they are now capable of handling very large biomedical datasets.

There are several potential advantages of linked data technologies and ontologies compared to classical technologies (e.g., non-semantic web pages, SQL databases, specialized REST and SOAP application interfaces). For example, it is now possible to create a decentralized network of diverse datasets that can be transparently queried through open web standards. Basic, machine and human-readable information about each entity can be retrieved through a simple HTTP GET request, thereby improving the transparency of large distributed datasets. The RDF/OWL standards can be used in multilingual environments. Powerful mechanisms for ontology-based alignment of data sources are also available.

However, user-friendly software applications based on linked data standards are still lacking. While there are several specialized and user-friendly interfaces for accessing certain linked datasets, such as a dedicated interface for aTags and a dedicated interface for the TCMGeneDIT data, there is a lack of good user interfaces for the exploration of aggregated and heterogeneous datasets. In our prototypical scenario, currently available, generic linked data browsers such as Marbles [35] or Sig.ma [36] did not produce a satisfactory user experience for ordinary pharmaceutical researchers. The linked data community must invest more resources in the creation of applications geared towards end-users. The creation of such applications may be simplified if linked data providers reuse existing upper ontologies and schemas, such as those offered by the Open Biological and Biomedical Ontologies (OBO) project [37].

## Concluding remarks

This article presents only the initial steps on a 'bridge' linking traditional medicines and modern pharmaceutical research. More of the existing databases about traditional medicines must be made publicly accessible and interlinked for broader integration. Semantic technologies and linked data provide a solid foundation for building such an integrated data infrastructure.

## Abbreviations

aTag: Associative tags (snippets of HTML that capture the information in a machine-readable, interlinked format); RDF: Resource description framework; SPARQL: SPARQL Protocol and RDF Query Language; OWL: Web Ontology Language; OBO: Open Biological and Biomedical Ontologies; TCM: traditional Chinese medicine

## Competing interests

The authors declare that they have no competing interests.

## Authors' contributions

MS wrote major parts of the article, implemented the aTag system and curated aTags from literature. MD created aTags about pharmacogenomic findings. JZ created the RDF conversion of TCMGeneDIT. JSL created prototypical implementations for identifying user-generated statements about efficacy and safety of traditional medicines from online discussion groups. MSM and KC provided creative input, support and guidance in the process of writing the article. All authors read and approved the final version of the manuscript.
